# Transmission patterns and evolution of respiratory syncytial virus in a community outbreak identified by genomic analysis

**DOI:** 10.1093/ve/vex006

**Published:** 2017-03-11

**Authors:** Charles N. Agoti, Patrick K. Munywoki, My V. T. Phan, James R. Otieno, Everlyn Kamau, Anne Bett, Ivy Kombe, George Githinji, Graham F. Medley, Patricia A. Cane, Paul Kellam, Matthew Cotten, D. James Nokes

**Affiliations:** 1 Epidemiology and Demography Department, Kenya Medical Research Institute (KEMRI) – Wellcome Trust Research Collaborative Programme, Kilifi, Kenya; 2 School of Health and Human Sciences, Pwani University, Kilifi, Kenya; 3 The Wellcome Trust Sanger Institute, Cambridge, UK; 4 Virosciences Department, Erasmus Medical Center, Rotterdam, The Netherlands; 5 Department of Global Health and Development, London School of Hygiene and Tropical Medicine, London, UK; 6 Virus Reference Department, Public Health England, London, UK; 7 Department of Infectious Diseases and Immunity, Imperial College London, London, UK; 8 School of Life Sciences and WIDER, University of Warwick, Coventry, UK

**Keywords:** full-genome sequencing, RSV, WAIFW, household transmission, community transmission

## Abstract

Detailed information on the source, spread and evolution of respiratory syncytial virus (RSV) during seasonal community outbreaks remains sparse. Molecular analyses of attachment (G) gene sequences from hospitalized cases suggest that multiple genotypes and variants co-circulate during epidemics and that RSV persistence over successive seasons is characterized by replacement and multiple new introductions of variants. No studies have defined the patterns of introduction, spread and evolution of RSV at the local community and household level. We present a whole genome sequence analysis of 131 RSV group A viruses collected during 6-month household-based RSV infection surveillance in Coastal Kenya, 2010 within an area of 12 km^2^. RSV infections were identified by regular symptom-independent screening of all household members twice weekly. Phylogenetic analysis revealed that the RSV A viruses in nine households were closely related to genotype GA2 and fell within a single branch of the global phylogeny. Genomic analysis allowed the detection of household-specific variation in seven households. For comparison, using only G gene analysis, household-specific variation was found only in one of the nine households. Nucleotide changes were observed both intra-host (viruses identified from same individual in follow-up sampling) and inter-host (viruses identified from different household members) and these coupled with sampling dates enabled a partial reconstruction of the within household transmission chains. The genomic evolutionary rate for the household dataset was estimated as 2.307 × 10 ^−^ ^3^ (95% highest posterior density: 0.935–4.165× 10 ^−^ ^3^) substitutions/site/year. We conclude that (i) at the household level, most RSV infections arise from the introduction of a single virus variant followed by accumulation of household specific variation and (ii) analysis of complete virus genomes is crucial to better understand viral transmission in the community. A key question arising is whether prevention of RSV introduction or spread within the household by vaccinating key transmitting household members would lead to a reduced onward community-wide transmission.

## 1. Introduction

Respiratory syncytial virus (RSV) is a leading viral cause of acute respiratory illnesses (ARI) worldwide (Haynes et al. 2013), with the virus infecting 5–10% of the world population annually (Falsey et al. 2005) resulting in an estimated 3 million hospitalizations of children aged under 5 years (Nair et al. 2010) and more than 160,000 deaths across all age groups each year (Nair et al. 2010). An important epidemiological feature of RSV disease is its highly seasonal patterns in communities (Stensballe et al. 2003). Globally, RSV disease occurs as recurrent annual epidemics that peak during the winter in temperate climatic regions but shows less consistent timing in the tropical or subtropical climatic regions (Stensballe et al. 2003; Haynes et al. 2013). No licensed RSV vaccine exists but several candidates are in development with some in phase three trials (Higgins et al. 2016). Infection prevention and treatment are currently limited to passive immunoprophylaxis, case isolation, and supportive care (Drysdale et al. 2016).

RSV belongs to family *Paramyxoviridae* and its genome is a non-segmented single-stranded negative-sense RNA molecule (∼15,200 nucleotides long) that encodes eleven viral proteins (in the order NS1-NS2-N-P-M-SH-G-F-M2 (1 and 2)-L). Two genetically and antigenically distinct RSV groups are recognized (A and B) whose local predominance alternates over successive epidemics (Mufson et al. 1985; Cane 2001, 2007). Based on phylogenetic analysis of the immunogenic and variable attachment (G) gene (Johnson et al. 1987), at least eight genotypes (and several variants within these genotypes) have been identified within each of the two groups (Peret et al. 1998, 2000; Agoti et al. 2015a). Analysis of RSV strains detected in several parts of the world found that RSV epidemics frequently comprise multiple genotypes (and variants) but locally a single genotype normally predominates an epidemic with periodic replacement in successive epidemics (Cane et al. 1992; Peret et al. 1998, 2000; Agoti et al. 2015a; Otieno et al. 2016). 

Improved understanding of RSV epidemiological patterns, transmission chains, and mechanism of persistence in host populations can help with infection control (Munywoki et al. 2014; Agoti et al. 2015a). Information on the origins of RSV seed strains for local epidemics, hubs of virus transmission, and spread patterns during outbreaks is limited (Nokes and Cane 2008; Munywoki et al. 2014; Agoti et al. 2015a). Detailed molecular analyses of RSV strains sampled during epidemics have the potential to elucidate these patterns (Agoti et al. 2015a and 2015b). However, such studies to date have primarily used samples collected from hospitalized individuals, representing a small and biased proportion (<1%) of all RSV infections during epidemics (Cane 2007). Community-based studies of RSV are rare (Munywoki et al. 2014). As a result, many aspects of RSV transmission, spread, and survival in the settings where majority of the infections occur remain unknown.

RSV surveillance in Kilifi County, located in coastal Kenya, has been ongoing since 2002 with a continuous hospital-based arm and intermittent community-based arm (Nokes et al. 2004, 2008, 2009; Munywoki et al. 2014; Agoti et al. 2015a]. Recently, we reported the RSV infection epidemiological findings from a cohort of forty-seven households followed over one epidemic season (Munywoki et al. 2014). Consistent with previous findings in developed countries (Hall et al. 1976) school-going children were found to be frequent introducers of the virus into households (Munywoki et al. 2014). Infection spread in the households was confirmed by group matching (typing into RSV A and B) and nucleotide comparison of the G gene (Munywoki et al. 2014). However, efforts to map transmission chains by combining the date of sampling and G sequence results showed limited success due to low phylogenetic signal from this short fragment (Munywoki 2013, 2014).

The intensive sampling regime during the household study provides an opportunity to uncover RSV transmission and evolution patterns in community epidemics. We recently showed that analysis of the relatedness of G gene sequences identified within and between epidemics can distinguish virus strains newly introduced into the community from those locally persisting (Agoti et al. 2015a). We also pointed out that a large fraction of RSV strains collected from local epidemics possess identical or highly similar G sequences (Agoti et al. 2015a; Zlateva et al. 2004; 2005). This illustrated the challenge of low phylogenetic resolution in undertaking detailed tracking of RSV transmission in a community by analyzing G gene sequences alone (Munywoki et al. 2014). However, when we compared full genomes of G identical strains, nucleotide differences were found occurring outside the G region (Agoti et al. 2015b). Thus, increasing the examined sequence length can provide much-needed additional phylogenetic resolution for monitoring virus transmission over short times (Cotten et al. 2013).

The analysis reported here investigated RSV A transmission in a community setting, the source of seed viruses and genomic diversification in a subset of samples collected during the household cohort study (Munywoki et al. 2014). We assessed the strength of the phylogenetic signal provided by analyzing the individual RSV genes versus for the whole genome sequences in tracking RSV transmission and the relatedness of the household viruses to contemporaneous strains across the world (Do et al. 2015). Further, due to the close monitoring of this cohort we were able to observe changes occurring at the consensus genome level intra- and inter-host during household transmission of RSV. In this report we show the utility of whole genome sequencing in defining RSV transmission, persistence, evolution and spread in households and at the local community level.

## 2 Materials and methods

### 2.1 Study location and population

**Figure 1.**
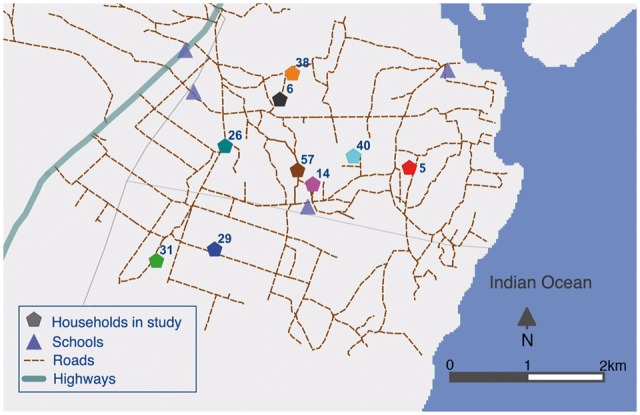
Geographical distribution of the nine studied households which each had at least one assembled genome. Also shown is the Mombasa-Malindi highway, roads and schools in the study area. Light grey lines indicate administrative sub-location boundaries.

The household study was undertaken within Kilifi County of Coastal Kenya in two local administrative units located to the north of the Kilifi Health and Demographic Surveillance System (KHDSS) (Scott et al. 2012). A household (HH) was defined as group of people living in the same compound and eating from the same kitchen (Munywoki et al. 2014). The area is primarily rural, with a number of small markets and the key economic activities include small-scale crop and animal farming, fishing and tourism. Overall, the county experiences a tropical climate with bimodal annual rainfall pattern: main rains April-July and shorter rains October-December. Annual RSV epidemics in this region, as recorded through surveillance in the Kilifi County Hospital (KCH), typically start in October-December of one year and continue to June-August of the following year (Agoti et al. 2015a; Nokes et al. 2009). The GPS locations of study households were recorded and entered in a confidential database. These addresses were validated in Google Earth and then visualized in QGIS v2.2 program (http://www.qgis.org/en/site/) overlaid with regional amenities data including local schools and main roads, Fig. 1. The sampled households occurred within an area of approximately 12 km^2^.

### 2.2 Study design

A detailed description of the household study design was provided in previous publications (Munywoki et al. 2014, 2015a, 2015b). Briefly, 47 households were recruited and closely followed up over a 6-month period between December 2009 and June 2010 to document all respiratory virus infection episodes. Twice weekly throughout the observation period, a nasopharyngeal-flocked swab was obtained from every household member regardless of the symptoms status. More than 80% of the planned samples were collected (Munywoki et al. 2014). The specimens were screened for a range of respiratory viral nucleic acids including RSV using multiplex real-time RT-PCR method (Gunson et al. 2005). A cycle threshold (Ct) of 35.0 or below was considered indicative of infection with the associated virus. In the current analysis all RSV A positive samples (187 RSV A mono-infected and 12 RSV A-B co-infected) from a select 13 households of the 47 were processed for whole genome sequencing and analysis, Table 1. These households were prioritized for analysis because RSV infection (group A or B) was detected in more than one member within a week suggesting a household RSV infection outbreak. The specimens had been collected between March and May of 2010 from 63 subjects. The arising sequence data were analyzed both independently and together with sequence data of RSV A strains from other countries deposited into GenBank.

**Table 1.** Characteristics of the households from which we analyzed RSV A positive samples and sequencing results.

HH ID	HH size	% Female	% In school	Median age (IQR) in years	Median number of samples (IQR)^a^	Number of RSVA Positive samples	Number of Genomes
5	37	64.9	24.3	11.4(3.3–23.5)	31(16–42)	70	24
6	6	100.0	50.0	11.4(1.9–16.5)	45.5(45–46)	2	1
12	20	50.0	30.0	16.6 (4.9–24.9)	24(11.5–40)	1	0
14	6	33.3	50.0	6.3 (2.8–9.4)	44.5(43–45)	18	12
19	14	57.1	50.0	13.0 (7.6–35.4)	41.5(34–43)	1	0
26	5	80.0	60.0	5.6 (2.7–11.5)	46(46–47)	9	9
29	7	42.9	42.9	7.9 (2.2–27.5)	43(42–43)	25	12
31	11	72.7	27.3	8.1 (2.3–27.6)	31(6–32)	11	5
38	23	43.5	43.5	12.6 (7.1–27.4)	40(36–43)	24	22
40	5	40.0	40.0	6.1 (2.0–8.9)	45(45–45)	12	10
45	10	70.0	80.0	11.4 (6.7–18.5)	42.5(31–45)	6	0
51	15	73.3	46.7	9.2 (3.3–28.4)	42(28–44)	2	0
57	16	43.8	50.0	12.9 (7.9–17.5)	28(21–29)	18	8

Abbreviations: HH for Household, ID for identity and IQR for interquartile range.

Near complete RSV genomes were obtained from only 9 of the 13 households we analyzed.

^a^ This refers to number of samples collected per a person in the respective households over the entire study period.

### 2.3 Ethics statement

The samples analyzed in this study were collected following an informed written consent from each individual participant if aged ≥18 years or through a guardian or parent if aged <18 years and all children assented to participate. The study protocol was reviewed and approved by both the Scientific and Ethics Review Unit (SERU) of the Kenya Medical Research Institute (KEMRI), Nairobi, and Coventry Research Ethics Committee, UK (Munywoki et al. 2014).

### 2.4 RNA extraction, amplification and sequencing

RNAs were extracted from raw nasal specimens using the QIAamp viral RNA extraction Kit following the manufacturer’s instructions (QIAGEN Ltd, London, UK). Complementary DNA (cDNA), PCR amplification and nucleotide sequencing of RSV genomes were performed as previously described (Agoti et al. 2015b). Briefly, the RSV genome was amplified as six overlapping fragments, which were henceforth pooled and used to prepare Illumina NGS libraries. These were subsequently sequenced using Illumina MiSeq, multiplexing 15 to 20 samples per run, to generate approximately 1-1.5 Million paired-end reads (150 bp × 2) for each sample.

### 2.5 Short read assembly into virus genomes

Raw sequence data from MiSeq were de-multiplexed into sample specific readsets and processed in QUASR (Watson et al. 2013) to remove low quality reads (median Phred score of <35) and primer and adapter sequences at the end of the individual reads. The resulting reads were *de novo* assembled using the SPades Program v3.5.0 (Bankevich et al. 2012) into contigs, examined for completeness of the expected open reading frames and, where necessary, partial contigs were further combined using Sequencher v5.0.1. To avoid errors due to crosstalk between multiplexed samples only contigs with a median read coverage of  > =500 were used. Genomes with gaps (< 500 nucleotides) were joined with a series of ambiguous nucleotides (Ns) using the most complete genome from the same household as a guide for inferring the length of the gap. Multiple Sequence Alignments (MSA) were generated in MAFFT v6.83 (Katoh et al. 2002).

Nucleotides at polymorphic positions on the genomes were checked as follows: A sequence alignment for each household was generated (all sequenced viruses) and any nucleotides showing variation from the group were directly examined. For each observed variant site, a 21-nucleotide (nt) motif spanning the variant nucleotide (normally at the center but adjusted for variants near the termini) was prepared. The frequency of these 21-mers (both forward and reverse complement sequences) in the quality-controlled short read data was then determined using a modified grep script Cartman.py (available at https://github.com/mlcotten/RSV_household_scripts) using ack (http://beyondgrep.com/why-ack/) and the majority nucleotide kept. In addition, all indels were directly examined and all ambiguous nucleotides (R, Y, S, W, M, K) were resolved by a similar direct read counting and with the ambiguous nucleotide replaced by the absolute majority nucleotide. In cases of a position having 2 or more variants with equal counts, the nucleotide variant present in the majority of the genomes from the study was used.

A total of 131 virus genomes for which the assembly yielded contigs >5000 nucleotides long were included in the analyses (i.e. gene-by-gene and whole genome analysis). These genomes were derived from 9 households. Of the 131 genomes, 103 were > 14000 nt in length with fewer than 500 ambiguous nucleotides (henceforth referred to as genomes, the only set considered in the whole genome analysis level). The alignment of the full genome was trimmed to include only sequence region covered by all genomes to maximize homology. The aligned sequences were analyzed for recombination using the RDP4 program and no recombination was detected (Martin et al. 2015).

### 2.6 Comparison dataset

Three data sets were prepared for comparison with the household study viruses. First, 11 G gene reference sequences, one for each of the known RSV A genotypes (GA1-7, SAA1-3 and ON1) were prepared and used for genotyping the household viruses on the basis of phylogenetic clustering. Second, 275 RSV A G sequences collated from GenBank that were sampled from different countries across the world between 2009 and 2010 and also from the Coastal Kenya in-patient surveillance at the KCH (Otieno et al. 2016) were prepared and used for determining the number and a probable source of the virus variants that seeded the household infection outbreaks. The third set included 354 nearly complete RSV A genomes retrieved from GenBank. These, inclusive of only genomes with information on country of origin, date of sampling and no recombination detected, were used to determine the global phylogenetic placement of the household viruses genomes.

### 2.7 Phylogenetic analysis

Phylogenies were generated from the nucleotide alignment of both whole genomes and from the excised individual genes. The trees were reconstructed using Maximum Likelihood (ML) method in either MEGA v5.22 (Tamura et al. 2011) or PhyML v3.1 program (Guindon et al. 2010). The best-fitted models of nucleotide substitution for each alignment were determined in IQ-TREE v1.4.3 (Nguyen et al. 2015). All gene-specific ML trees were inferred in MEGA under HKY85 model bootstrapping for 1,000 replicates. Whole genome ML trees were inferred in PhyML v3.1 under GTR + Γ_4_ model of substitution, with 1,000 bootstraps. A bootstrap value of >70% was considered as statistically significant.

The potential transmission networks within and between households were inferred in PopART package v1.7.2 (http://popart.otago.ac.nz/index.shtml). The networks were reconstructed using median joining trees (MJT) method with an epsilon of zero.

### 2.8 Genotyping, variant and cluster analysis

The household viruses were genotyped by phylogenetic clustering pattern of their G ORF region with reference G sequences. Representative sequences of all known RSV A genotypes (GA1-7 & ON1) were included. A genome was assigned to a particular genotype if its G sequence clustered with the genotype reference sequence within the same branch with > 70% bootstrap support. To understand the evolution and transmission history of the identified viruses within the same genotype, the sequences were further typed into variants. Viruses were defined as same variant if their divergence was estimated to have occurred no more than a year before their date of collection and this helped identify independent virus introductions into the study area. We inferred these by considering the number of nucleotide differences observed in the G ectodomain region for virus pairs as recently described elsewhere (Agoti et al. 2015a). This method asserts that 4 or more nucleotide differences between viruses in the G ectodomain indicates a distinct virus variant, a criterion that takes into consideration the fragment length, substitution rate and time interval between the samples (Agoti et al. 2015a). The number of variants was also confirmed by the relatedness of the household viruses in the presence of contemporaneous background diversity from multiple countries across the world (Agoti et al. 2015a). A cluster was defined as a group of viruses that do not meet the distinct genotype or variant threshold rules but fall within one tree branch with a bootstrap support of > 50%.

### 2.9 Evolutionary analyses

The temporal signal in nucleotide divergence of the household viruses was estimated in TempEst v1.4 (Rambaut et al. 2016) using a ML whole genome tree as input. The evolutionary pattern and time to the Most Recent Common Ancestor (tMRCA) of the obtained whole genome sequences were determined in BEAST v1.8.2 under HKY85 model of substitution, (uncorrelated) lognormal relaxed molecular clock and Gaussian Markov random field (GMRF) population skyride (Minin et al. 2008; Drummond and Rambaut 2007; Drummond et al. 2012). The Metropolis Coupled Markov Chain Monte Carlo (MC-MCMC) chain length was set to 50 Million steps sampling after every 2500 steps. The output was examined in Tracer v1.6 (http://tree.bio.ed.ac.uk/software/tracer/), with a 10% burn-in removal, to confirm run convergence (i.e. if the estimated sample size for all inferred parameters was >200). The output trees were summarized in TreeAnnotator (Drummond and Rambaut 2007) (with a 10% burn-in removal) and the resulting Maximum Clade Credibility (MCC) tree was visualized and annotated in FigTree v1.4.2 (http://tree.bio.ed.ac.uk/software/figtree/). A posterior probability of > 0.9 was interpreted as statistically significant.

### 2.10 Sequence nomenclature and accession numbers

The sequence nomenclature on the phylogenetic trees is country of origin (_sample source for Kilifi indicating if sampled from inpatient (IP) or household (HH))/Unique identifier/Date of specimen collection. The unique identifier for household samples includes the household identifier (first two digits) and subject identifier (the last two digits). All new sequences from this study were deposited in GenBank under the accession numbers KX510136-KX510266.

## 3 Results

### 3.1 Genome alignment, genotyping and variant analysis

**Figure 2.**
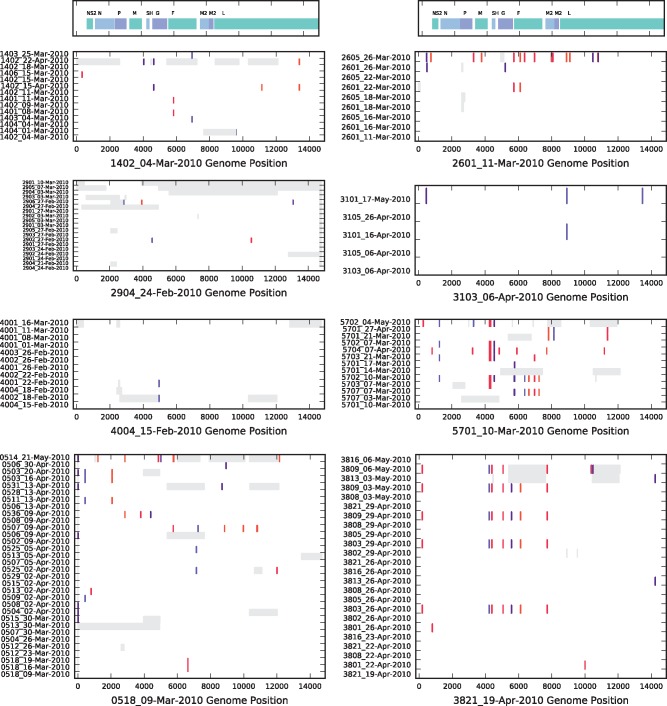
Nucleotide differences between viruses (total = 130) detected within the individual households. Each panel is a single household. The viruses were compared to the earliest virus genome sequenced from the same household. Vertical colored bars show the nucleotide differences. Red is a change to T, orange is a change to A, purple is a change to C and blue is a change to G. Grey is a deletion or an non-sequenced portion of the genome. Household six is excluded as only a single genome sequence was obtained. A python script to generate this figure is available at https://github.com/mlcotten/RSV_household_scripts.

**Figure 3.**
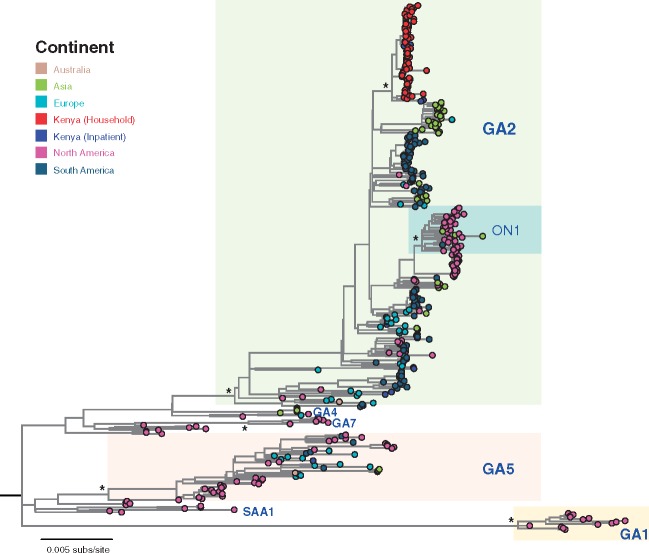
A ML inferred phylogenetic tree showing the global phylogenetic context of the RSV A household study genomes. The taxa of the household study viruses (*n*= 103) are in red while viruses from the rest of Kenya (inpatient) are colored blue. The taxa of RSV A viruses from around the globe are colored by continent of origin. Asterisk mark has been placed next to major branches with a bootstrap support of >70%.

The baseline characteristics of the households yielding RSV A positive samples and details on the number of genomes obtained per household are given in Table 1. Nucleotide changes were observed across the entire RSV genome (Fig. 2) in the 8 households with more than one genome sequenced. Within individual households, the number of nucleotide changes between virus genomes was variable and ranged from 0-17 nucleotides. Of the 131 specimens yielding contigs of >5000 nt, 120 from 10 households yielded an intact G coding sequence (CDS) and all these belonged to genotype GA2 and the closely related sub-genotype NA1 (result not shown). These household genomes formed a single monophyletic group within genotype GA2 on the global phylogeny (Fig. 3) that was most closely related to GA2 genotype viruses from Coastal Kenya that had been sampled from young children admitted to KCH in the years 2009 and 2010 [15]. Further, the entire set of RSV A viruses from the households fell within a single variant definition as also determined by their clustering of the G gene genomic region in the global G-gene phylogeny (Supplementary Fig. S1).

### 3.2 Relatedness and phylogeny of the household viruses

**Figure 4.**
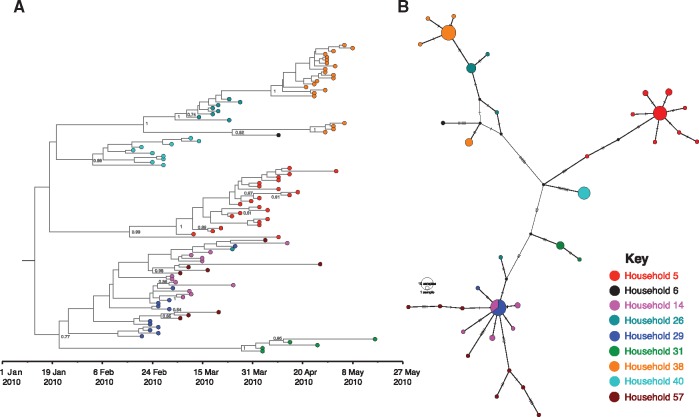
The sequence relatedness of the household study RSV A viruses. (a) A time-scaled phylogenetic tree of the 103 genome sequenced household study viruses inferred in BEAST program. The genomes are represented by a filled circle colored differently for each household (color scheme similar to Fig. 1). (b) A median-joining (MJ) haplotype network constructed from the 103 household genomes. Each colored vertex represents a sampled viral haplotype, with different colors indicating the different households of origin. The size of the vertex is relative to the number of sampled isolates. Hatch marks indicate the number of mutations along each edge. Small black circles within the network indicate unobserved internal nodes.

A time-resolved phylogenetic clustering of the 103 household study genomes (Fig. 4, panel A) revealed that all viruses clustered by household of origin, except for those from households 26, 38 and 57. This pattern was also observed with a ML phylogeny (Supplementary Fig. S2) and MJT network that showed household-specific clustering of viruses as well as a varied level of the interconnection of viruses within and between households (Fig. 4, panel B). Viruses from households 5, 31 and 40 formed individual distinct household-specific clusters that included all virus genomes obtained from these households. In contrast, households 26, 38 and 57 had genomes from 2 or more separate branches, suggesting multiple virus introductions into each of these three households. Particularly in household 26, three virus genomes from individual 2605, collected on the 16^th^, 18^th^ and 22^nd^ March clustered with the other viruses from that household (Supplementary Fig. S2). However the virus genome obtained from 26^th^ March appeared on a lone branch suggesting a second introduction of a genetically varied virus. Genomes from households 14 and 29 were interspersed within the same viral cluster. Household 6 provided only one genome.

In contrast to the genome-based phylogeny, when considering individual gene ORFs, the resolution was reduced and fewer household-specific distinct clusters were identified compared to the full genome analysis. ML phylogenetic clustering of the sequenced viruses by ORF is shown in Supplementary Fig. S3 (whole genome phylogeny included for comparison purposes, panel xi). When we considered the G gene alone (901 nt), just one household had a distinct virus cluster (HH 31); the remaining clusters included viruses from multiple households. Similarly reduced resolution was obtained with the F gene (1727 nt) with only two household-specific clusters (HH 6 and 40), the nucleoprotein (N) gene (1200 nt, with also only two household-specific clusters (HH 5 and 40) and with the L gene (7915 nt), four household-specific clusters were observed (HH 5, 6, 31 and 40). For comparison, the full genome analysis showed seven household specific clusters.

### 3.3 Between households transmission

The spatial distribution of the nine households is shown in Fig. 1. The geographical distance between the study households ranged from 302 to 3925 meters. There were a variable number of nucleotide differences across the genomes distinguishing clusters of viruses found in one household from the next (range 2-16), Fig. 4, panel B. The RSV A infection was first detected in household 40 (on 15^th^ February) followed by 29 (21^st^ February), 14 (1^st^ March), 57 (3^rd^ March), 5 (9^th^ March), 26 (11^th^ March), 31 (30^th^ March), 6 (9th April) and finally household 38 (19^th^ of April). For some of the study households, the infection periods overlapped. Notably, both HH 14 and 57, being the closest households in geographical distance (∼300 meters apart), had the first RSV infections detected in the first week of March (2 days apart) and virus strains were phylogenetically close when compared to strains from most other households we analyzed (Fig. 4 and Supplementary Fig. S2). This scenario was also observed with HH 6 and 38 (∼400 meters apart). Although these two cases were consistent with the hypothesis that physical distance modulates virus transmission and spread, there were household pairs that showed a contrary relationship, for example some members of household 14 and 29 gave multiple identical full genome sequences despite the two households being 1715 meters apart. Statistical analysis of the entire household dataset did not find a linear relationship between physical and genetic distance for this dataset (R^2 ^=^ ^0.01686).

### 3.4 Within-household transmission and sequence variation

**Figure 5.**
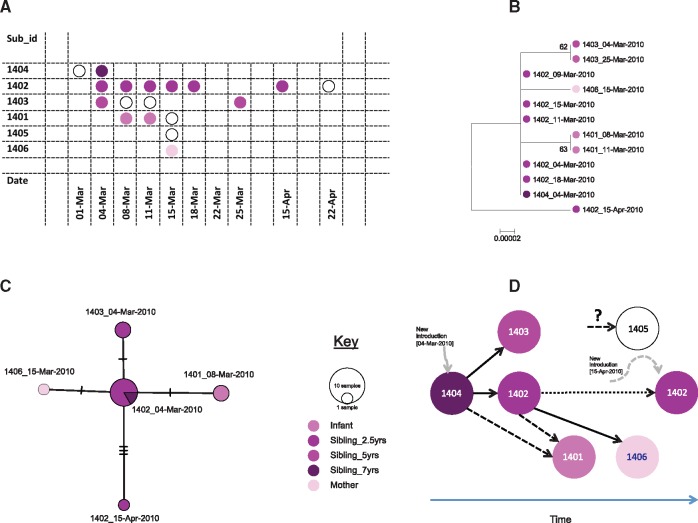
Inferred virus transmission patterns within household 14. (a) Temporal infection patterns. Every rectangular box represent a sample collected from members of the household 14, if there is a circle inside implies the sample was RSV A positive. Unfilled circle implies specimen was not sequenced while filled colored circle implies sample was sequenced (whole genome). (b) A ML phylogenetic tree from whole genome sequences of 12/18 sequences sequenced. Same circle color for sample from the same individual. (c) A median joining haplotype network of 12 genomes. Each vertex presents a sampled viral haplotype, with different colors indicating different individuals who provided the sample. The size of the each vertex is relative to the number of sampled isolates. Hatch marks indicate the number of mutations along each edge. (d) The putative inferred transmission events. Continuous arrow indicates where the transmission link was inferred as highly likely while dotted arrows indicate where multiple alternative scenarios could have been the source of infection.

We reconstructed a plausible virus transmission chain between the household members by combining the genetic data with sampling dates. As examples we show analysis for HH 14, a six-member household (Fig. 5) and household 38, a 23-member household (Supplementary Fig. S4). In household 14, of the 18 RSV positive samples identified in this household, 14 assembled into contigs >5000nt and 12 gave near complete genomes. From the sample collection dates, we inferred that the individual designated 1404 introduced the virus into this household since this individual was the only virus positive person in this household on the 1^st^ March (Fig. 5, panel A). Subsequently, the other household members designated 1401, 1402 and 1403 became virus positive within a week after the identification of individual 1404 RSV positivity. The genome data were consistent with individual 1404 (index case) infecting individuals 1402, 1403 and 1401 being identical or displaying only one nucleotide difference across their genomes, Fig. 2, Panel C. Each of the individuals 1405 and 1406 had both only a single virus positive sample collected on 15^th^ March (two weeks after first sample from the index case). Sequencing was unsuccessful with the sample from individual 1405. However, the sample from 1406 had one or two nucleotide changes compared with all genomes in this household. The virus from individual 1406 was genetically closest to virus from individuals 1402 and 1404 but it is more likely that 1406 acquired the infection from individual 1402 who showed prolonged virus shedding. It is also important to note that some viruses identified in household fourteen were identical to those observed in household twenty-nine thus we could not exclude a second introduction of the virus into this household.

Individual 1402 was virus positive for the longest period (39 days) compared to other members in this household, Fig. 5, Panel A. Interestingly, the positive sample collected on the 15^th^ April came after several samples collected between 20^th^ March and 13^th^ April had tested RSV negative. The virus from 1402 on 15^th^ April had 3 nucleotide substitutions that distinguished it from all the other viruses sampled from this household, Fig. 5, panels B and C. This scenario could have arisen due to: (i) another virus introduction into the household or (ii) a virus rebound (recrudescence) from initial infection in this individual after accumulating these changes. Combining the genome sequence and temporal diagnostic information we inferred the transmission chain presented in Fig. 5, panel D, for this household.

### 3.5 tMRCA, evolutionary rates, amino acid changes

TempEst analysis estimated that the MRCA for the household viruses occurred in December 2009 and their evolutionary rate was 4.948 ×10 ^−^ ^3^ sub/site/year. Notably, the R squared value for the linear model was 0.29 indicating the stochastic nature of variation observable in this limited time period. Different households had differing levels of diversity with only limited temporal relationship to this variation (Supplementary Fig. S5). Using BEAST program, the date of the MRCA for the household dataset was estimated to be 3^rd^ Jan 2010 (95% HPD: 1^st^ November, 2009 to 31^st^ Jan, 2010), corresponding to the beginning of the Kilifi 2009/10 RSV epidemic season. This date was consistent with a single virus variant leading to the RSV A infections in all nine analyzed households. The BEAST-inferred genomic evolutionary rate for the household viruses was estimated as 2.307 × 10 ^−^ ^3^ (95% HPD: 0.935× 10 ^−^ ^3^ to 4.164 × 10 ^−^ ^3^) sub/site/year. This was about 5 fold higher compared to previous estimates for data derived across epidemics (Agoti et al. 2015b). While synonymous nucleotide (dS) changes were found in RSV encoded proteins, non-synonymous nucleotide (dN) changes were observed in only 7 of the 11 RSV proteins (NS2, SH, G, F, M2-1, M2-2, L) with the highest number of dN changes observed in the L protein region (11 independent changes). The NS1, N, P and M were totally conserved at the amino acid sequence level. A summary of the amino acid changes observed between the household genomes for all the ORFs are shown in Table 2. The F protein had the third highest number dN changes (most of these affecting 27-mer amino acid domain (pep27)). Changes in the G protein were spread throughout its length but outside of the central conserved cysteine noose region. All the household genomes contained six highly conserved N-glycosylation sites within their F protein, at positions 27,70,120, 126 and 500. Also six completely conserved N-glycosylation positions were found within the G protein: 85, 103, 135, 251, 273, and 294. All the household viruses were observed to encode uniform F and G protein lengths, 574 and 297, respectively.

**Table 2.** Amino acid changes in the household viruses’ genomes by encoded protein.

NS-1	NS-2	N	P	M	SH	G	F	M2-1	M2-2	L
	I43M				E2G	L13P	I59V	V125I	Y36C	S224N
	R50G					N34H	F114S		N40Y	N236D
						I49T	P119L		N44K	K591N
						P143L	G130S		I83T	I955I
						P146L	V516A			N970K
						T148A	T529I			T1045M
						D214E				T1174S
						T268A				I1588T
										E1619G
										L1746S
										Y1762F

## 4 Discussion

Our knowledge of RSV transmission in the community, evolutionary patterns and ‘who acquires infection from whom’ (WAIFW) is incomplete (Agoti et al. 2015a; Munywoki et al. 2014). Close contacts within households, workplaces, worship places, market places and other social gathering avenues may provide opportunities for respiratory virus transmission (La Rosa et al. 2013). However, there is little evidence beyond temporal patterns of case occurrence to support that households are a major environment of RSV transmission (Munywoki et al. 2014; Hall et al. 1976). Viral genetic data can provide evidence to support epidemiological linkage of household RSV infected cases and to discount other sources of the infection.

Our findings support the hypothesis that RSV transmission within households is common as members belonging to the same household were infected with closely related strains, in terms of genomic sequence than viruses found in members from different households. Specifically, household-specific genomic variation was observed in seven of the nine households where we compared associated genomes. Only two households shared a genetically identical strain at full genome level. Notably, this between-household phylogenetic resolution was lost when examining the individual genes (including the G gene), as genetic variations between the sequenced viruses were random and distributed throughout their entire RSV genomes such that examining greater sequence lengths linearly increased the phylogenetic resolution achieved.

The genomes of all the household study viruses fell within a single branch on the global phylogeny and G gene analysis suggested that all the nine households were infected by a single virus variant that had entered into this community. Due to limited contemporary sequences from other parts of Kenya or Africa, it was not possible to identify close ancestors of this variant (Agoti et al. 2015a). Furthermore, it was not possible to infer the directions of household-to-household transmissions or pathway of the spread of infections reported here, because only a minority of the households in the study area were sampled. However, some of the households that were physically close happened to be infected by viruses that were also phylogenetically close. This is consistent with the idea that occupants of neighboring households are more likely to come into close contact during daily activities for example journeys to fetch water, to markets and clinics. It is also more likely that children in physically close households go to the same school, which are thought to be respiratory virus transmission hubs.

Within two individual households (HH 38 and 57), we observed higher genomic variation. We hypothesize three possible sources for this variation: (1) multiple virus introductions into these households, (2) co-infection of the index case with multiple genetic variants, and (3) diversification of a single virus in the process of replication and transmission through the members of the households. Some of the households had clearer evidence of multiple virus introductions (e.g. household fifty-seven) and this may be a result of factors that cannot be comprehensively investigated from our limited sampling. However, further analysis of these data including inspection of the minor variant populations is necessary to provide additional illumination (Hughes et al. 2012; Grad et al. 2014; Do et al. 2015). It is also possible that some of the observed changes simply reflected PCR and/or sequencing errors. However this is highly unlikely especially where nucleotide changes were observed at the same exact genomic position in multiple samples from the same household or individual despite their independent sample processing (Cottam et al. 2008). Also, importantly, only contigs with high read depth (> = 500) were included into our analysis.

The variation of genomes within households aided in identifying members who are likely to have shared an infection source or sequentially transmitted the infection from one to the other (e.g. the chains inferred for household fourteen and thirty-eight). However, it was not possible to elaborate in complete detail the transmission chains within most households even after considering these genomic data. This was partly due to incomplete sequencing (some samples had too low virus load) and also due to fact that the evolutionary rate of the virus was sometimes too low to provide a useful signal. This is likely to be caused by the highly infectious nature of RSV once introduced into a household setting resulting in overlapping infection generations before distinct nucleotide changes accumulate.

The evolutionary rates calculated at genome level from the household outbreak were significantly higher than rates derived from long-term data (Tan et al. 2012, 2013; Agoti et al. 2015b;). Our findings support the notion that evolutionary rates for viruses are highly context-specific and decrease when calculated from long-term sampling data (Duchene et al. 2014). This may reflect that deleterious mutations occurring during short-term transmission (and observed in the higher frequency sampling) that are purified from the virus population in the longer term. Multiple nucleotide changes were observed across RSV genome but some genes remained completely conserved at the amino acid sequence level. Although it is unlikely that the amino-acid substitutions observed represented adaptive evolution during short-term transmission of the virus, it will be worthwhile to further investigate their significance in allowing virus survival or escape from pre-existing immune responses.

Among respiratory viruses, viral genetic data have been previously utilized for influenza A viruses to define within and between household virus spread. Sequencing of hemagglutinin and neuramidase genes of 2009 pandemic H1N1 viruses found occurrence of only limited genetic diversity for viruses derived from different households early during the outbreak and diversity was negligible for viruses derived from same households (Thai et al. 2014). Deep sequencing of household viruses from Hong Kong revealed that genetic variation was more similar within than between households and associated information on minor variant sharing helped confirm transmission events (Poon et al. 2016).

For RSV, our study is the first of its kind using full genomic data to define patterns of its transmission in a community setting. Using temporal infection data alone, it has been previously concluded that young children are most likely to introduce RSV infection into households (Hall et al. 1976; Munywoki et al. 2014; Heikkinen et al. 2015) and the genetic data provided here support this conclusion. Within household RSV transmission has never been inferred to the detail described here. The evidence of multiple virus introductions in some households was particularly intriguing and would been missed if partial sequencing alone was deployed. Our study shows that patterns of shared virus strains between households can vary by the gene analyzed, but it is possible to separate almost all households as infected by a distinct virus strain by analyzing full genome sequences.

We are aware of limitations in this study. First, sampling in the households only reached ∼85.6% of the planned level with gaps mostly occurring in adults (Munywoki et al. 2014). Thus, it is possible that we missed important samples in inferring the transmission chains. Second, a significant proportion (34.2%) of the samples failed amplification, especially those with low viral load, hampering the reconstruction of transmission chains. However, this difficulty is common to all such studies (Memish et al. 2014; Bose et al. 2015 ). Third, PCR and sequencing errors were not completely modeled into the interpretation of our data (Orton et al. 2015). Despite our analytical stringency, it is possible that some of the nucleotide changes we observed could be artifacts especially those occurring in single genomes only. Fourth, we only analyzed a small proportion of households in the study area and important information such as contact patterns and school attendance were not factored into the analysis. This made it difficult to infer the broader community transmission pathways and exclude multiple sources of identical virus into a household.

In conclusion, our study has shown that the analysis of genome sequences provides better phylogenetic resolution in tracking RSV spread compared to analysis of small partial sequences including the highly variable G gene. Although whole genome analysis alone could not resolve every step in the transmission chains within households, the information derived distinguished many of the between-household transmission links and suggested clear epidemiological linkage of infections of some household members. The findings are consistent with a large percentage of RSV transmissions occurring within the household and thus infection control at the household level should be considered in RSV disease control. Future studies should include mathematical modeling to combine whole genome analysis (both consensus and minor variants data) with other epidemiological information (e.g. symptoms onset, viral load, immunity, social contact patterns, etc.) to allow mapping of WAIFW with regard to RSV spread within households.

## Supplementary Material

Supplementary Data

## Data Availability

All sequence files are available from the GenBank database (accession numbers KX510136-KX510266). For more detailed information beyond the metadata used in the paper, there is a process of managed access requiring submission of a request form for consideration by our Data Governance Committee (http://kemri-wellcome.org/about-us/#ChildVerticalTab_15).
