# Supplementary material for: Transmission patterns and evolution of respiratory syncytial virus in a community outbreak identified by genomic analysis
Source: Virus Evol. 2017 Mar 11;3(1):vex006. doi: 10.1093/ve/vex006 (PMC5399923; doi:10.1093/ve/vex006)
Supplement: Supplementary Data [file vex006_Supp.pdf]

Supplementary material

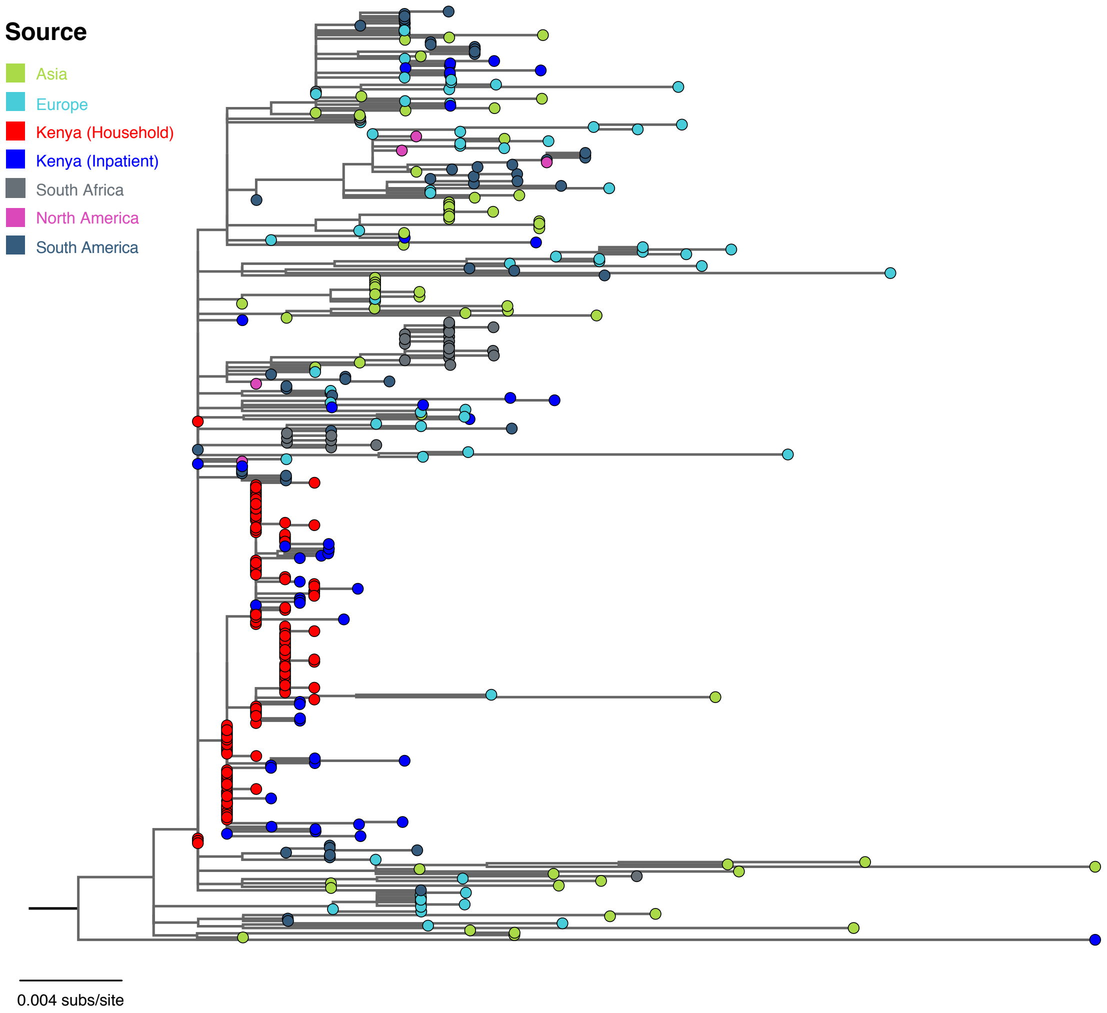

Fig. S1. A G-gene based ML inferred phylogenetic tree showing the clustering of the household study with global RSV A viruses sampled in the years 2009 and 2010. The taxa of the household study viruses are in red while viruses from the rest of Kenya are colored blue. The taxa of RSV A viruses from other included countries from around the globe are colored by continent of origin.

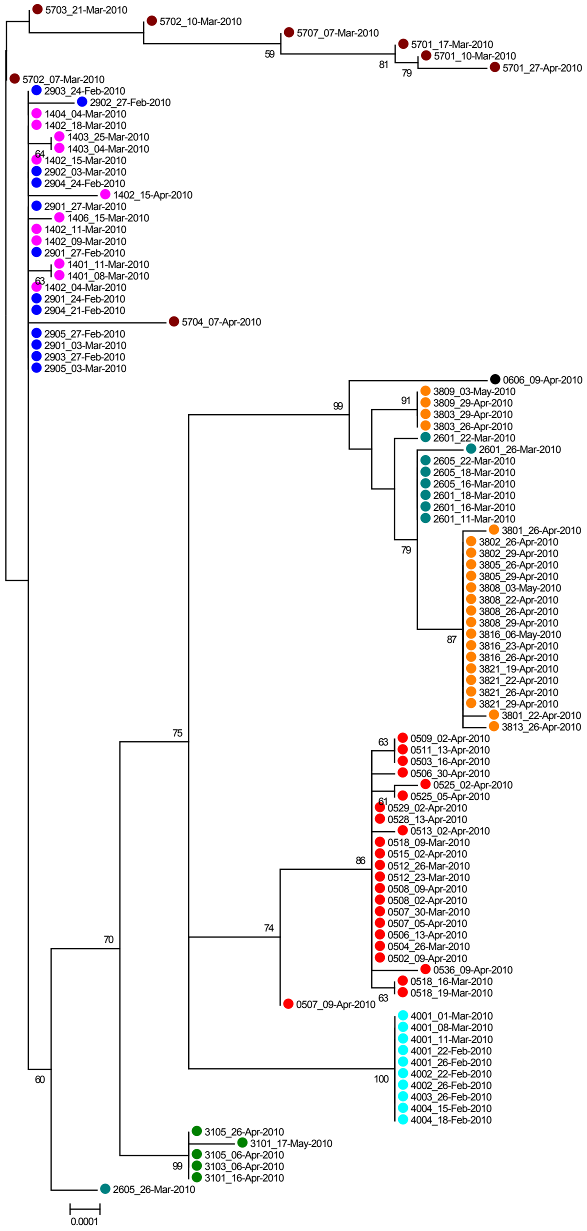

Fig. S2 A ML inferred phylogeny of household study genomes (n=103). The taxon names include the household identifier (first two digits) and subject id (the last two digits). The phylogeny taxon names a proceeded by a filled circle colored differently for each household with a color scheme similar to Fig. 1 and 4. The tree is mid-point rooted.

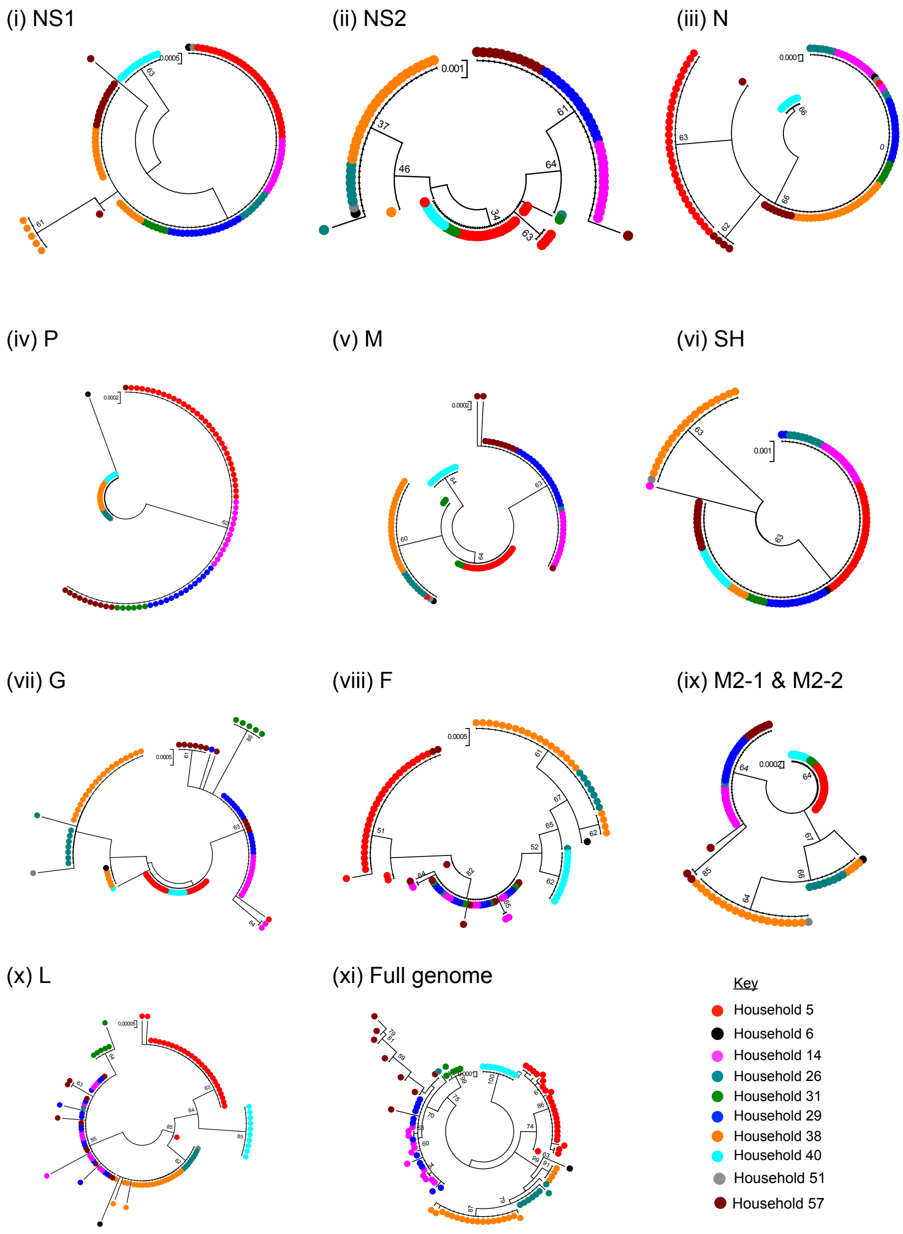

Fig. S3 The ML phylogenetic clustering of the household study viruses as observed in the analysis of different ORFs. The taxa of the viruses are colored differently for each household. The colour scheme is as in Fig. 1 and 4. For comparison purposes the whole genome phylogeny had included (panel xi).

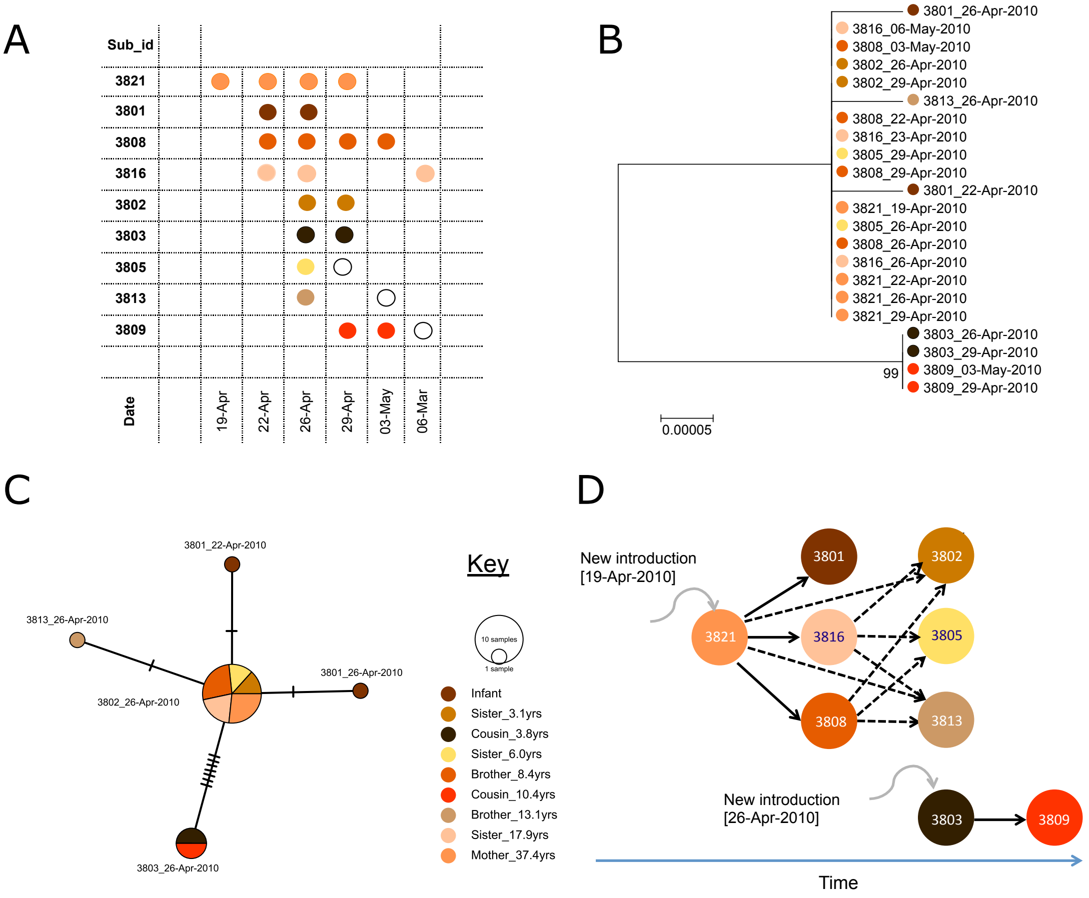

Fig. S4 Inferred virus transmission patterns within household 38. Panel A. Temporal infection patterns. Every rectangular box represent a sample collected from members of the household 38, if there is a circle inside implies the sample was RSV A positive. Unfilled circle implies specimen was not sequenced while filled colored circle implies sample was sequenced (whole genome). Panel B. A ML phylogenetic tree from whole genome sequences of 12/18 sequences sequenced. Same circle color for sample from the same individual. Panel C. A median joining haplotype network of 22 genomes. Each vertex presents a sampled viral haplotype, with different colors indicating different individuals who provided the sample. The size of the each vertex is relative to the number of sampled isolates. Hatch marks indicate the number of mutations along each edge. Panel D. The putative inferred transmission events. Continuous arrow indicates where the transmission link was high inferred as highly likely while dotted arrows indicate where multiple alternative scenarios could have been the source of infection.

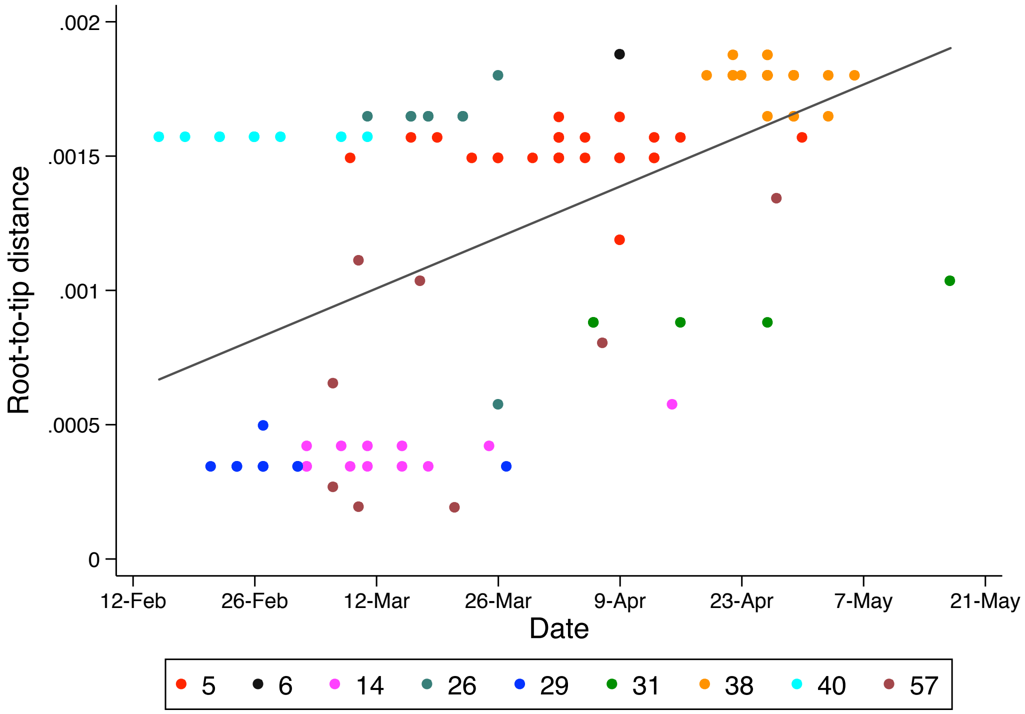

Fig. S5 A regression of the root-to-tip genetic distances in the maximum-likelihood phylogenetic tree of the 103 respiratory syncytial virus household genomes. The distances were inferred in TEmpEst program. The different data point colors reflect the different households of origin and the color scheme is as in Fig. 1, Fig. 4 and S2 Fig. The equation for the regression line is y = 0.00005x - 0.5248 with a R^2 of 0.290.
